# Inferring Balancing Selection From Genome-Scale Data

**DOI:** 10.1093/gbe/evad032

**Published:** 2023-02-23

**Authors:** Bárbara D Bitarello, Débora Y C Brandt, Diogo Meyer, Aida M Andrés

**Affiliations:** Biology Department, Bryn Mawr College, Bryn Mawr, Pennsylvania; Department of Integrative Biology, University of California, Berkeley, Berkeley, California; Department of Genetics, Evolution and Environment, UCL Genetics Institute, University College London, London, United Kingdom; Institute of Biosciences, Department of Genetics and Evolutionary Biology, University of São Paulo, São Paulo, SP, Brazil; Department of Genetics, Evolution and Environment, UCL Genetics Institute, University College London, London, United Kingdom

**Keywords:** natural selection, population genomics, summary statistics, composite likelihood ratio tests, genetic variation, heterozygote advantage

## Abstract

The identification of genomic regions and genes that have evolved under natural selection is a fundamental objective in the field of evolutionary genetics. While various approaches have been established for the detection of targets of positive selection, methods for identifying targets of balancing selection, a form of natural selection that preserves genetic and phenotypic diversity within populations, have yet to be fully developed. Despite this, balancing selection is increasingly acknowledged as a significant driver of diversity within populations, and the identification of its signatures in genomes is essential for understanding its role in evolution. In recent years, a plethora of sophisticated methods has been developed for the detection of patterns of linked variation produced by balancing selection, such as high levels of polymorphism, altered allele-frequency distributions, and polymorphism sharing across divergent populations. In this review, we provide a comprehensive overview of classical and contemporary methods, offer guidance on the choice of appropriate methods, and discuss the importance of avoiding artifacts and of considering alternative evolutionary processes. The increasing availability of genome-scale datasets holds the potential to assist in the identification of new targets and the quantification of the prevalence of balancing selection, thus enhancing our understanding of its role in natural populations.

**Significance**

Natural selection is a fundamental mechanism of evolution that plays a crucial role in shaping and maintaining adaptive traits within populations. Balancing selection is a specific form of natural selection that maintains genetic diversity within a population, rather than favoring a single trait or allele. Understanding the extent to which variation within populations is influenced by balancing selection, as well as the loci and traits it affects, is a significant challenge in the field of evolutionary biology. In this review, we examine current methodologies that use genomic data to identify regions of the genome that are evolving under balancing selection. These approaches have the potential to reveal a significant number of previously unknown loci under balancing selection. As a result, we are on the brink of having a greatly enhanced understanding of the loci influenced by balancing selection, as well as the timing of this process.

## Definitions and Challenges

**Fig. 1.**
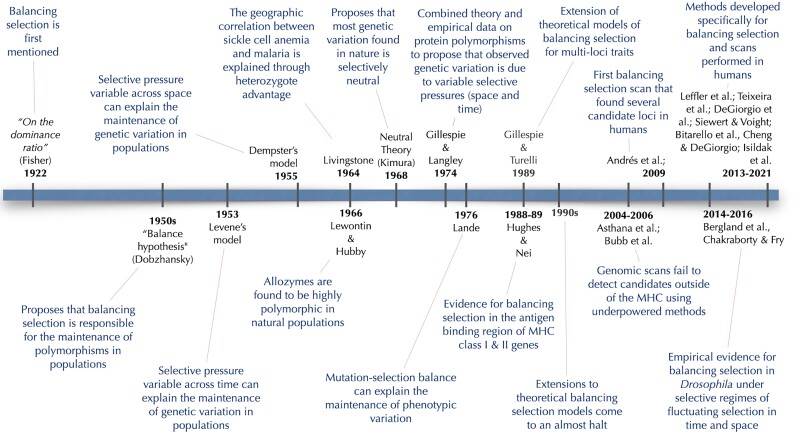
BLS timeline. References in the figure: Fisher (1922); Levene (1953); Dempster (1955); Dobzhansky (1955); Livingstone (1964); Hubby and Lewontin (1966); Kimura (1968); Gillespie and Langley (1974); Lande (1976); Hughes and Nei (1988, 1989); Asthana et al. (2005); Bubb et al. (2006); Andrés et al. (2009); Leffler et al. (2013); Bergland et al. (2014); DeGiorgio et al. (2014); Teixeira et al. (2015); Chakraborty and Fry (2016); Siewert and Voight (2017, 2020); Bitarello et al. (2018); Cheng and DeGiorgio (2019, 2020, 2021); Isildak et al. (2021).

Balancing selection (BLS) is natural selection that maintains advantageous genetic and phenotypic diversity in populations (Lewontin 1987). Its name traces back to the influential “balance hypothesis,” which predicted that natural populations would harbor extensive levels of genetic diversity, with natural selection being responsible for actively maintaining different alleles “in balance” (Dobzhansky 1955). The dichotomy between the balance hypothesis and the “classical” hypothesis—whereby purifying selection would result in low levels of diversity—dominated the field of population genetics for decades. Following the proposal of the neutral theory of molecular evolution and subsequent theoretical perspectives that accommodated a range of selective regimes of varying intensities, the notion that BLS provides a comprehensive explanation for overall genetic diversity lost much of its relevance. Figure 1 shows landmarks in our understanding of BLS.

Though no longer seen as the primary process maintaining genetic diversity, empirical and theoretical interest in understanding BLS has persisted. It is well appreciated that BLS explains polymorphism and adaptive phenotypic diversity at various loci and species and is responsible for the presence, in some loci, of hundreds of variants (reviewed in Meyer et al. 2018). Moreover, there is an increasing appreciation that BLS plays a role in fundamental biological processes such as sex determination (Charlesworth 2004), self-incompatibility (Lawrence 2000), and immune response (Andrés et al. 2009; Bitarello et al. 2018). BLS can also shape the evolution of phenotypes directly related to survival, with recent studies illustrating that genome-wide heterozygosity may predict adult survival (Doyle et al. 2019; Scott et al. 2020). By maintaining adaptive polymorphisms in populations, BLS is likely to maintain genetic diversity that contributes to evolutionarily relevant phenotypes, including those seen as diseases in extant populations (Carter and Nguyen 2011).

A central challenge to BLS studies and the development of tests designed to identify genomic regions evolving under this selective regime is that it is, in fact, an amalgam of selective mechanisms that maintain beneficial diversity in the population (table 1). These include heterozygote advantage or overdominance, negative frequency-dependent selection, antagonistic selection (including sexually antagonistic selection or antagonistic effects in different tissues or over the lifespan), and selection that changes across time or space in a panmictic population. Two textbook examples illustrate the diversity of mechanisms and timescales of BLS: the maintenance of the *S* mutation in the hemoglobin B locus (*HBB*) in sub-Saharan human populations and the extensive polymorphism of the genes within the major histocompatibility complex (MHC) (table 2).

**Table 1:** Diverse Mechanisms of BLS

Mechanism	Definition	Theoretical Predictions
Heterosis or heterozygote advantage	Heterozygous genotype(s) have higher fitness than homozygotes.	Excess of heterozygotes. Shifted SFS toward frequency equilibrium, typically observed as an excess of intermediate-frequency alleles (not necessarily 0.5). Excess of polymorphism if selection is sufficiently old.
Negative frequency-dependent selection	Fitness is a function of allele frequency in the population. Includes rare allele advantage, which preserves polymorphism because fitness increases when the allele is rare.	Shifted SFS toward the frequency equilibrium, typically observed as an excess of intermediate-frequency alleles (not necessarily 0.5). For rare allele advantage, excess of low-frequency alleles instead. Excess of polymorphism if selection is sufficiently old.
Antagonistic selection (including sex, tissue, or lifespan components)	The fitness of alleles differs across conditions, for example, between sexes or over the lifespan.	Shifted SFS toward the frequency equilibrium, typically observed as an excess of intermediate-frequency alleles (not necessarily 0.5). Excess of polymorphism if selection is sufficiently old. Different allele frequencies in different conditions (e.g., males/females, at different ages) if selection affects survival.
Selection that varies across space in a panmictic population	Heterogeneity of the selection coefficient across a species or population range leads to the maintenance of alleles that are beneficial in only some locations.	Shifted SFS toward intermediate-frequency alleles (not necessarily 0.5). Excess of polymorphism if selection is sufficiently old. Different allele frequencies in different locations. Possible deficit of heterozygotes given the allele frequencies.
Selection fluctuates over time in a panmictic population	Heterogeneity of selection coefficient across time.	Shifted SFS toward intermediate-frequency alleles (not necessarily 0.5). Excess of polymorphism if selection is sufficiently old. Different allele frequencies at different time points.

SFS, site frequency spectrum.

**Table 2:** Examples of BLS Timescales in Humans

Timescale	Dates	Examples
Ultra long-term	$>7\times 10^{6}$ years ($T_{div}+4N_{e}$ generations)^a^	MHC locus (Klein et al. 1998), ABO blood group locus (Ségurel et al. 2012)
Long-term	$>10^{6}$ years ($>4N_{e}$ generations)^b^	MHC locus (Garrigan and Hedrick 2003), *ERAP2* (Andrés et al. 2010)
Recent	$<10^{6}$ years	*HBB-S* (Laval et al. 2019), *MEFV* (Isildak et al. 2021)

a $\;T_{div}+4N_{e}$ generations is the expected coalescence time between lineages present in species that diverged $T_{div}$ generations ago. $T_{div}$ between humans and chimpanzees is approximately 6 million years and generation time is approximately 25 years.

b $4N_{e}$ generations is the expected TMRCA in a genealogy under neutrality. In humans, the long-term effective population size ($N_{e}$) is about $10^{4}$ individuals.

The *HBB-S* mutation in humans attains frequencies of up to 11% in regions of Africa where malaria is endemic, despite the markedly reduced fitness of homozygous individuals for this mutation—who have sickle-cell disease. Overdominance at this locus (Livingstone 1964) results from heterozygotes developing blood cells that efficiently transport oxygen while being resistant to infection by the malaria parasite, *Plasmodium falciparum*. Here, selection favors a specific heterozygous genotype, is geographically delimited to regions where malaria is endemic, and begins around 20,000 years ago when increased sedentarization of human populations provided favorable conditions for the proliferation of the malaria parasite (Laval et al. 2019) (table 2).

Selection on MHC loci, on the other hand, is documented in numerous species (Radwan et al. 2020). In humans, human leukocyte antigen (HLA) genes show evidence of selection starting several million years ago, as indicated by the sharing of polymorphisms between humans and other primates (Leffler et al. 2013; Teixeira et al. 2015), with divergence times exceeding 6 million years (table 2). Although the specific selective mechanisms maintaining HLA diversity remain under debate, most models include host–pathogen co-evolution, with pathogen mutations that allow evasion from the immune response exerting pressure for compensatory changes in host immunity (Meyer et al. 2018; Ebert and Fields 2020; Radwan et al. 2020).

The contrast between the well-known examples of the *HBB* and MHC loci illustrates the diversity of selective regimes, timescales, and biological processes underlying BLS. Here, we will focus on the inference of BLS from genome-scale data and will not attempt to detail the underlying mechanisms (but see table 1 for a summary) since several excellent reviews exist (Hedrick 2012; Key et al. 2014; Fijarczyk and Babik 2015). Instead, we will focus on the genomic footprints of BLS and on how we can identify them and use this information for biological inference, with examples.

Several factors have recently brought a renewed interest in methods to identify the genomic targets of BLS. First, years of research showed the great potential of genomic methods to identify targets of positive selection in genomes, even in challenging scenarios—for example, very recent timescales and selection from standing variation (Messer and Petrov 2013). This generated a growing interest in identifying targets of other types of natural selection, including BLS, which had remained comparatively unexplored. Second, population-level genome-scale sequencing is becoming available for many species. Dense SNP data generated by sequencing efforts are necessary because the genomic signature of BLS is often much narrower than that of positive selection. The lack of dense SNP data likely contributed to early genome-wide studies failing to detect BLS (Asthana et al. 2005; Bubb et al. 2006). Further, as discussed later, the density of polymorphisms is itself a BLS signature, making full sequencing data necessary. Third, several methods to study BLS benefit from interspecies comparisons, and the expansion of population genomic studies beyond model organisms contributes to the utility of these approaches. Finally but critically, recent studies claim that the number of loci under BLS may be much greater (Bitarello et al. 2018; Soni et al. 2022) than previously thought (Asthana et al. 2005; Hedrick 2012).

A well-supported list of loci evolving under BLS is crucial to understanding its relevance in the evolution and genetic diversity of populations. How prevalent is BLS in natural populations? How long can balanced alleles survive? Which genes and genomic elements have evolved under BLS? Are there biological functions that are particularly prone to evolve under BLS? Does BLS contribute to disease in extant populations? Answering these questions ultimately demands methods that rely on genomic data—discussing them is the primary goal of this review.

## Timescales of Balancing Selection

**Fig. 2.**
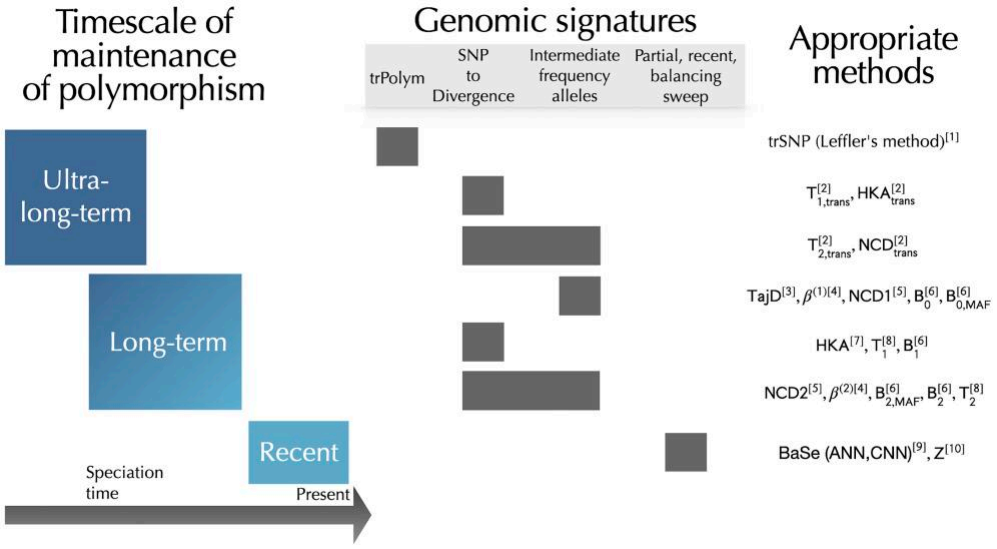
Timescales and genomic signatures of BLS and methods that can detect them. References in the figure: [1] Leffler et al. (2013); [2] Cheng and DeGiorgio (2019); [3] Tajima (1989); [4] Siewert and Voight (2017); [5] Bitarello et al. (2018); [6] Cheng and DeGiorgio (2019); [7] Hudson et al. (1987); [8] DeGiorgio et al. (2014); [9] Isildak et al. (2021); [10] (Soni et al. 2022). In humans, the ranges are roughly defined as follows (see table 2): recent ($<10^{6}$ years); long-term ($>10^{6}$ years); ultra long-term ($>7\times 10^{6}$ years).

Although biologically distinct, the different mechanisms of BLS (table 1) result in genomic signatures that are often indistinguishable. One convenient consequence is that common BLS signatures—high polymorphism and an excess of alleles at intermediate frequencies—characterize several BLS mechanisms, thus guiding many strategies designed to identify BLS targets. Unfortunately, attributing these signatures to any particular mechanism of BLS is thus challenging and requires careful consideration. The opposite is true regarding the selection timescale, as different ages of selected alleles generate distinct signatures and require different methods (fig. 2). The timescale boundaries are not clear-cut and depend on effective population size and demographic history. However, because the selection timescale is an essential aspect of the inferred selective regime, we discuss the detection of BLS as a function of the age of onset of selection at a given locus. Table 2 summarizes these boundaries for humans, along with examples.

### Long-Term Balancing Selection (LTBS)

Alleles under BLS have a reduced probability of being fixed or lost compared to those evolving neutrally or under directional selection (Maruyama and Nei 1981; Takahata 1990; Schierup et al. 2000); consequently, they tend to be older than other polymorphisms. We will refer to BLS that is old and persistent enough to maintain polymorphism longer than expected under neutrality as *long-term balancing selection* (LTBS). In humans, this corresponds to a timescale in the order of millions of years (table 2), given by $4N_{e}$—the mean (or expected) time to the most recent common ancestor (TMRCA) for neutral alleles.

#### Unusually Old TMRCA and Increased Levels of Polymorphism

Because alleles under LTBS fix at lower rates, multiple neutral or nearly neutral polymorphisms accumulate in their linked genomic region (Maruyama and Nei 1981; Takahata 1990; Schierup et al. 2000; Charlesworth 2006). Thus, a high level of polymorphism due to long TMRCA is a prime signature of LTBS. Most—if not all—BLS mechanisms result in increased levels of polymorphism: overdominance (Kaplan et al. 1988), negative frequency dependence, temporally fluctuating selection (Huerta-Sanchez et al. 2008), spatially fluctuating selection (Levene 1953), and antagonistic pleiotropy (Connallon and Clark 2013). Thus, methods based on this signature can detect a range of mechanisms and, consequently, many selection targets.

On the other hand, an old-time to TMRCA is not universal: selection may be recent or may not drastically increase the TMRCA of linked variants—for example, in the case of fluctuating selection (Taylor 2013). Nevertheless, increased polymorphism due to old TMRCA is highly specific for LTBS. In the absence of BLS, it can only be generated by admixture or introgression (table 3), which leaves additional signatures of long-range linkage disequilibrium (LD) (Charlesworth et al. 1997; Racimo et al. 2015). Of note, Bitarello et al. (2018) found that few putative BLS targets overlap known Neanderthal introgressed segments suggesting that, at least for methods that can identify narrow signatures of increased polymorphism (as all methods to identify BLS should), this confounding factor does not result in substantial levels of false positives. Excess polymorphism is thus a signature ubiquitously used to identify LTBS.

**Table 3:** Nonselective Mechanisms Can Originate Patterns Similar to Those of BLS

Pattern of Variation	Major Nonselective Process that can Originate It	References
Local excess of heterozygotes (deviation from HWE)	Technical artifacts due to mapping errors in genomic regions with paralogy	Meyer et al. (2018)
SFS with excess intermediate-frequency alleles	Recent bottlenecks, admixture or substructure	Nielsen (2005)
Excess of polymorphism	Low levels of introgression or admixture and technical artifacts	Charlesworth et al. (1997); Racimo et al. (2015); Setter et al. (2020)
High polymorphism and low differentiation	Local adaptation followed by gene-flow	Jin et al. (2022)

HWE, Hardy–Weinberg equilibrium; SFS, site frequency spectrum.

#### Skewed Allele Frequency Spectrum

Another signature of LTBS is a distortion in the site frequency spectrum (SFS). Under several mechanisms and conditions of BLS—for example, under overdominance or antagonistic pleiotropy (Carter and Nguyen 2011)—the selected and linked alleles can be maintained at or near an equilibrium frequency—that is, the frequency that maximizes mean fitness in the population. The effect on linked (primarily neutral) variants generates a signature characterized by an SFS with an excess of intermediate-frequency alleles compared with what would be expected under neutral evolution (Charlesworth 2006). Under neutrality, a similar skew in the SFS could only be generated by recent population bottlenecks, admixture or substructure, which would affect the genome as a whole, not specific loci as in the case of BLS (table 3).

Other mechanisms may not lead to a stable equilibrium—for example, negative frequency dependence, whereby genotype fitness changes with allele frequency, and temporally fluctuating selection, whereby fitness changes over time or seasonally/cyclically—but will still generate an SFS shifted to intermediate-frequency alleles. In these cases, polymorphism can be maintained even when heterozygote fitness is not the highest, so long as the geometric mean heterozygote fitness exceeds that of the homozygotes (Gillespie 1973; Felsenstein 1976; Ginzburg 1977; Wittmann et al. 2017). An excess of intermediate-frequency alleles is thus a prevalent signature of LTBS. Under conditions such as symmetric overdominance, where both alleles (assuming a bi-allelic locus) have the same fitness, the equilibrium frequency is 0.5. Most often, however, the equilibrium frequency is different from 0.5. Accordingly, a key novelty of recent approaches is their acknowledgment that BLS can generate distortions of the SFS with an excess of alleles at frequencies other than 0.5 (Siewert and Voight 2017, 2020; Bitarello et al. 2018).

We note that not all mechanisms of BLS share this signature. For example, fluctuating selection with cyclical fluctuations in fitness may generate large numbers of low-frequency alleles or a U-shaped SFS (Huerta-Sanchez et al. 2008) and not be captured by methods focused on an excess of intermediate-frequency alleles—although it could still be identified by methods that detect increased levels of polymorphism.

#### Other Signatures

Additional allele-frequency-based signatures can be generated by BLS, although they are often harder to identify and differ among selective mechanisms. Sexual antagonism can generate different allele frequencies in males and females (Wang et al. 2022) if selection acts on survival (Kidwell et al. 1977); temporarily fluctuating selection can generate allele frequency change over time (Bergland et al. 2014; Wittmann et al. 2017); spatially fluctuating selection can generate geographic frequency differences in panmictic populations (Levene 1953; Hedrick 2006; Chakraborty and Fry 2016); overdominance and sexually antagonistic selection (Zajitschek and Connallon 2018) can generate an excess of heterozygotes in relation to Hardy–Weinberg expectations (HWE) (Mead et al. 2003).

### Ultra Long-Term Balancing Selection (ULTBS)

LTBS can maintain functional polymorphisms for remarkable amounts of time. Although uncommon, such loci are among the best-known examples of BLS. In primates, the MHC (Klein et al. 1998) and the ABO blood group loci (Ségurel et al. 2012) exemplify how polymorphisms can be maintained in multiple species for millions of years (table 2). Similar cases exist in other species, including sex-determining systems (Hasselmann and Beye 2004) or self-incompatibility loci in plants (Lawrence 2000). Particularly striking is that these cases represent selection that has been strong and stable enough to overcome drift over millions of years, even after inevitable changes in demography and environment. We will refer to BLS that is old and constant enough to be shared across species as *ultra long-term balancing selection* (ULTBS, fig. 2). For a human–chimpanzee comparison, this corresponds to roughly $7\times 10^{6}$ years (table 2).

What could explain the selection persistence over extended periods if environments are rarely constant? The answer is perhaps that environmental change is in itself the cause of selection. A prime example is that of immune-related loci. While responding to a specific pathogen may be transiently adaptive, long-term adaptation may be better defined as the “ability to respond to changing pathogens” (Ebert and Fields 2020)—while the repertoire of pathogens is dynamic, the selective pressure on immune genes is continuous. The selective pressure need not be external: in self-incompatibility polymorphisms—which maintain variation that limits self-fertilization—the selection is constantly driving an avoidance of inbreeding, but there is a turnover of the specific alleles involved in incompatibility. These examples highlight that conditions that favor ULTBS can be achieved even with changing environmental conditions.

ULTBS can originate trans-species polymorphisms (trPolym)—that is, alleles shared across species and inherited from their common ancestor. With sufficient divergence time, the sharing of neutral polymorphisms among species is so unlikely that their presence is a signature of ULTBS (Leffler et al. 2013; Gao et al. 2015; Teixeira et al. 2015). For example, given known mutation rates, the probability that a neutrally evolving SNP in humans will also be polymorphic in chimpanzees is $1.6\times 10^{-8}$, and of additionally being polymorphic in bonobos is $4\times 10^{-10}$ (Teixeira et al. 2015). Several studies have used true shared SNPs between humans and chimpanzees and suggested that ULTBS in humans is rare (Leffler et al. 2013; Gao et al. 2015; Teixeira et al. 2015), at least insofar as shared trPolym can be detected. The term “true” is critical here as most polymorphisms shared among species are not inherited from a common ancestor and thus not trPolym but recurrent mutations or sequencing errors.

While instances of trPolym provide convincing evidence of ULTBS, not all cases of ULTBS are expected to result in a trPolym. If old balanced polymorphisms are lost in one (or both) species, but selective pressures remain and new balanced polymorphisms arise, no trPolym will exist. Even if the trPolym is missing in the genomic dataset, and even though the long-term effects of recombination results in narrow genomic signatures (Charlesworth 2006), the signatures of ULTBS surrounding such a locus resemble those of LTBS, although theoretically with a higher excess of polymorphism due to an older TMRCA (Cheng and DeGiorgio 2019). This property can and has been explored by some methods, as we will discuss.

### Recent Balancing Selection (RBS)

When selected alleles are not distinctively older than neutral alleles, we refer to *recent balancing selection* (RBS, fig. 2). In humans, we define this as less than 1 million years (table 2). Many recent balanced alleles will not survive long because selective pressures are neither stable nor strong enough to maintain them long-term. Nevertheless, the many young, short-lived balanced polymorphisms may have significant collective phenotypic effects even if each one has weak fitness effects—which we expect if transiently balanced alleles are under weaker selection than stable ones.

The unremarkable TMRCA means that the most characteristic signature of LTBS and ULTBS—increased diversity—is absent. RBS is characterized by a rapid frequency increase of a beneficial allele (possibly the youngest of the two balanced alleles), and, fundamentally, the process is equivalent to that of a recent, partial sweep of positive selection (Charlesworth 2006; Isildak et al. 2021), with the difference that the allele frequency increase halts once the equilibrium frequency is reached. The corresponding genomic signature is thus not unique to RBS, hampering the identification of its targets with LTBS methods. RBS due to overdominance can theoretically generate an excess of heterozygous genotypes compared with HWE expectations, although this requires extreme selective pressures in the current generation. Moreover, one must ensure that departures from HWE are not due to technical artifacts of the polymorphism data (table 3). Not surprisingly, few cases have been documented. Low differentiation of allele frequencies among populations is another potential signature of RBS, if selection slows down the rate of population divergence under drift, making selected markers distinguishable from those evolving neutrally (Lewontin and Krakauer 1973). This signature will arise if the BLS regime is shared among populations and the selected allele is the same (Brandt et al. 2018).

## Methods to Identify LTBS

Until recently, no methods were explicitly tailored to detect BLS. Nevertheless, classic neutrality tests were used for this purpose—mainly Tajima's *D* test (Tajima 1989), the Hudson-Kreitman-Aguadé (HKA) test (Hudson et al. 1987), or the Ewens-Watterson homozygosity (EWH) test (Watterson 1978). With the recent availability of population genomic data, we have seen a plethora of methods developed to detect signatures of LTBS, with higher power than the classic approaches. This improved our ability to identify LTBS targets but requires choosing the appropriate test. We review available methods according to the selection timescales, the target signature, and data requirements (tables 4 and 5). We focus on modern rather than classical methods and divide them into those based on summary statistics and model-based composite likelihood ratio tests (CLRTs).

**Table 4:** Available Software that Implements Tests for BLS

Software	Methods or Tests Implemented	URL
Betascan (python)	$\beta^{(1)*}$ ; $\beta^{\left(1\right)}$; $\beta_{std}^{(1)*}$; $\beta^{\left(2\right)}$; $\beta_{std}^{\left(2\right)}$	https://github.com/ksiewert/BetaScan
BALLET (C)	$T_{1}$ ; $T_{2}$	http://degiorgiogroup.fau.edu/ballet.html
NCD-Statistics (R)	NCD1;NCD2	https://github.com/bbitarello/NCD-Statistics
balselr (R)	NCD1;NCD2	https://github.com/bbitarello/balselr
MuteBaSS (python)	$NCD2_{trans};\;NCD2_{mid,trans};\;$ $NCD2_{opt,trans};\;HKA_{\;trans}$	https://github.com/bioXiaoheng/MuteBaSS
MULLET (C)	$T_{1,trans}$ ; $T_{2,trans}$	http://degiorgiogroup.fau.edu/mullet.html
BalLeRMix (python)	$B_{0}\;;B_{0,MAF}$ ; $B_{1};\;B_{2};\;B_{2,MAF}$	https://github.com/bioXiaoheng/BalLeRMix
BalLeRMix + (python)	$B_{0};\;B_{0,MAF}$ ; $B_{1};\;B_{2};\;B_{2,MAF}$	https://github.com/bioXiaoheng/BallerMixPlus
BaSe (python)	Artificial and convoluted neural networks.	https://github.com/ulasisik/balancing-selection
ANGSD	Tajima's *D*; Fu & Li's *F*; Fu & Li's *D*; Fay's *H*; Zheng's *H*	https://github.com/ANGSD/angsd
tskit (python)	Tajima's *D*	https://github.com/tskit-dev/tskit
Tsel (R)	$T_{sel}$	https://blogs.cornell.edu/clarklabblog/clark-lab/software
C code	HKA	https://github.com/alanrogers/hka
C code	HKA	https://github.com/andrewkern/hka
C code	HKA	https://bio.cst.temple.edu/!∼tuf29449/hka_manual

This list is by no means exhaustive and focuses on software that implements tests discussed here.

**Table 5:** Recent Methods Aimed at or Frequently Used to Detect Signatures of LTBS and ULTBS at Individual Loci

Methods or Tests	Input Data Required		
	Ingroup Species	Outgroup Species	Information on Ancestral State Needed?
$T_{1},\;B_{1}$	Number of polymorphic sites	One reference sequence to infer divergence	No
$HKA_{trans},T_{1,trans}$ ^a^	Number of polymorphic sites	Number of polymorphic sites	No
$NCD1,\;\beta^{(1)*},\;\beta_{std}^{(1)*},B_{0,MAF}$	Minor allelic frequencies	–	No
$NCD2,\;B_{2,MAF}$	Minor allelic frequencies	One reference sequence to infer divergence	No
$\beta^{\left(1\right)},\;\beta_{std}^{\left(1\right)},\;B_{0}$	Derived allelic frequencies	One reference sequence to infer the ancestral state^b^	Yes
$\beta^{\left(2\right)},\;\beta_{std}^{\left(2\right)},\;T_{2},\;B_{2}$	Derived allelic frequencies	One reference sequence to infer divergence	Yes
$NCD2_{trans}$	Minor allelic frequencies	Minor allelic frequencies	No
$T_{2,trans}$	Derived allelic frequencies	Derived allelic frequencies	Yes
trPolym	High-quality sequence data for several individuals	High-quality sequence data for several individuals	No

^a^For these tests, the number of ingroup species is flexible, and the required data applies to all ingroup species.

^b^If the ancestral state is known, no other information from an outgroup is required.

Neutrality tests relying on summary statistics reduce one or more aspects of the genetic data to a single number. While there is a loss of information inherent to summarizing complex patterns in a single statistic, their major advantages are ease of implementation and interpretation, speed, well-established expected behaviors under a myriad of demographic models, and, often, relative robustness to mis-specified demographic models.

CLRT methods—which involve explicit modeling of neutral expectations and application of a likelihood ratio test to compare the likelihoods under null and selection models—can offer more power by effectively integrating information across sites in a genomic region rather than summarizing them in a single value. CLRT methods often have increased power compared to summary statistics but at the cost of computational time, memory usage, and, critically, a heavy reliance on model assumptions (e.g., known demographic history of the population). They are thus most useful for well-studied populations and species.

Because exploring the entire space of model parameters in a likelihood framework is intractable for complex models, some implementations use precomputed scenarios (e.g., DeGiorgio et al. 2014). Another approach to circumvent the difficulty of exact likelihood computing for complex selection models is approximate Bayesian computation (ABC). With ABC, the observed data is compared with millions of simulations under the reasonable assumption that simulations that best fit the observed data, based on several summary statistics, best reflect the selective history of a locus. ABC applied to specific genes or genomic regions can help infer the most likely selection models and parameters (Peter et al. 2012; de Filippo et al. 2016). While ABC has been successfully used for continuous parameter inference (e.g., effective population sizes, and selective coefficients), it is not optimal for joint estimation of continuous and categorical parameters (e.g., neutrality vs. selection), as discussed in Sheehan and Song (2016).

### Methods to Detect Higher-Than-Expected SNP Density

An excess of polymorphism to substitution (the latter being used to control for heterogeneity in mutation rate) has long been used to identify BLS targets, traditionally by the HKA test (Hudson et al. 1987). The HKA test contrasts the number of variable sites within one population (polymorphisms) or between a pair of species (substitutions) to the expected numbers under neutrality. Andrés et al. (2009) proposed HKA_LOW_, a one-tailed interpretation of the HKA test, which only tests for an excess of polymorphism (vs. substitutions), and later methods used similar approaches (e.g., Cheng and DeGiorgio 2019). HKA and related tests rely on correctly estimating the neutral background variation, which entails modeling to determine which summary statistic values are extreme under neutrality. Alternatively, neutral expectations can be inferred from the empirical genomic distribution assuming that a small portion of the genome evolves under BLS. Thus, loci with the most extreme summary statistic values are prime candidate targets. These two strategies to identify putative target loci are commonly used with other summary statistics as well.

The CLRT statistics *T*_1_ and *T*_2_ (tables 2 and 3) implemented in the software BALLET (DeGiorgio et al. 2014) (table 2, fig. 2) explicitly model the probability of a site being polymorphic in a given species, given that it is either a polymorphism or a substitution. These probabilities are derived from the coalescent process of a site under BLS following the model by Kaplan et al. (1988). Briefly, given an ingroup species and an outgroup species (e.g., humans and chimpanzees, respectively) and the expected genome-wide interspecies coalescence time, we can infer the likelihood of an informative site being a polymorphism (or a substitution) under both BLS and neutrality. The neutral estimate is computed directly from the genome-wide SFS and thus accounts for demographic factors that may affect the genome globally. $T_{1}$ is tailored to detect an excess of polymorphism relative to divergence (substitutions) and is therefore analogous to the summary-based HKA test—but significantly outperforms it (DeGiorgio et al. 2014; Siewert and Voight 2017; Bitarello et al. 2018; Cheng and DeGiorgio 2020). Modified versions implemented in the software MULLET (table 4)— $T_{1,trans}$ and $T_{2,trans}$ (Cheng and DeGiorgio 2019)—extend this framework to an arbitrary number of ingroup species for cases where BLS is shared across species (table 5).

The *B* statistics (Cheng and DeGiorgio 2020)— $B_{0},\;B_{0,MAF},\;B_{1},\;B_{2,MAF},\;B_{2}$ —implemented in BalLerMix (table 4) are also CLRT methods. Among them, $B_{1}$ is an analog of HKA and $T_{1}$ because it takes into account substitutions and polymorphisms but not allele frequencies (table 5). The model underlying the *B* statistics approximates the probability of observing a given number of alleles at a particular frequency equilibrium in a balanced locus as following a binomial distribution with *n* trials (the haploid sample size) and success rate *x* (the expected derived or minor allele count, depending on the method). The model approximates the probability of a neutral site being under BLS as following an exponential decay with increasing recombination distance from the site under selection. Thus, sites far from the target site are down-weighted, reducing noise in the estimate. The probability of a neutral site having *x* derived or minor allele counts is approximated by the binomial, the genome-wide SFS, or a mixture of both.

### Methods to Detect a Skewed SFS

The SFS may be analyzed using either the frequency of derived alleles (DAF) in the form of an unfolded SFS or using minor allele frequencies (MAF) in the form of the folded SFS. While the use of unfolded SFS sometimes improves power over the folded SFS (Siewert and Voight 2017, 2020; Cheng and DeGiorgio 2020), in practice, the ancestral state is not always known as it requires an outgroup species (table 5).

Tajima's *D* (*TajD*) test, a classic summary of the SFS, uses as a statistic the difference between two estimators of θ for a sample of DNA sequences—one based on the number of segregating sites ($\theta_{W}$), the other based on the number of pairwise differences ($\theta_{\Pi }$). *TajD* remains commonly used in BLS studies in nonhuman species (table 4). We note, however, that there are many better-powered methods available (DeGiorgio et al. 2014; Siewert and Voight 2017, 2020; Bitarello et al. 2018; Cheng and DeGiorgio 2019, 2020).

Another classical test is the EWH test (Watterson 1978), which captures both excess and reduced homozygosity compared to neutral expectations, making it nonspecific to BLS, as is the case for *TajD*. Andrés et al. (2009) proposed a test specific to shifts in the SFS as expected under BLS: MWU_HIGH_, a Mann–Whitney one-tailed test that tests for differences between the SFS of a given locus and its expectation under neutrality (e.g., as obtained from the genome-wide empirical distribution).

Modern summary statistics explicitly tailored to detect shifts in the SFS due to LTBS include $\beta^{(1)\;}$ (Siewert and Voight 2017) and NCD1 (Bitarello et al. 2018) (table 4). Both exploit the expectation that neutral variation linked to sites under LTBS will have allelic frequency close to that of the balanced polymorphism. $\beta^{\left(1\right)}$ captures the correlation in allelic frequency between a core SNP (the SNP assumed to be under LTBS) and nearby neutral SNPs by contrasting two population mutation rate parameters: $\theta_{W}$ (Watterson's estimator) and $\theta_{\beta }$ (the mean similarity of allele frequencies between each linked SNP and the core SNP, excluding the core SNP) for a given window. More specifically, $\beta^{\left(1\right)}=\theta_{\beta }-\;\theta_{W}$, where $\theta_{\beta }=\frac{\sum_{i=1}^{n-1}\;id_{i}S_{i}}{\sum_{i=1}^{n-1}\;d_{i}}$, for all *i* SNPs in a window except the core SNP, where $S_{i}$ measures the DAF for site *i* across a sample of chromosomes, and $d_{i}$ measures the similarity in DAF between each SNP in a window and the core SNP (Siewert and Voight 2017). $\beta^{\left(1\right)}$ can thus be interpreted as a weighted average of SNP counts based on their similarity to the core SNP.

NCD1 measures the mean squared difference between a target MAF (*tf*, target frequency) and the frequency of each tested SNP, analogous to a standard deviation that measures dispersion not from the mean of the distribution, but from *tf*, the expectation under BLS (noncentral deviation, *NCD*). The lower the *NCD*, the higher the proportion of sites with allele frequencies close to the *tf*, and the stronger the signature of selection. NCD1 requires predefining a putative frequency of the selected allele *tf*, which is typically unknown. However, *NCD* correlates highly across *tf* values, so its choice does not influence results strongly and this shortcoming can be addressed by using multiple *tf* values (Bitarello et al. 2018).

Both $\beta^{(1)*}$ and NCD1 share the strength of being easy to implement and not requiring knowledge about ancestral states: NCD1 is explicitly based on the folded SFS and $\beta^{\left(1\right)}$ (which uses the unfolded SFS) offers a folded ($\beta^{(1)*}$) implementation and, as noted by the authors, the signature captured by $\beta^{\left(1\right)}$ is not influenced by whether nearby neutral variants are linked to the ancestral or derived allele (Siewert and Voight 2017) (table 5). NCD1 and $\beta^{\left(1\right)}$ also have several differences. For NCD1 all polymorphisms are equally weighted and the target frequency is defined based on the assumed deterministic equilibrium frequency of the target SNP. For $\beta^{\left(1\right)}\text{and }\beta^{(1)*}$, the similarity of the allele frequency (DAF or MAF, respectively) to that of the core SNP is raised to a power set by the parameter *p*, which controls to what degree linked alleles contribute to the statistic: p=0 entails all variants in the window are weighted equally and $\theta_{\beta }=\theta_{W}$ and when p→∞ only variants that have precisely the same frequency as the core SNP contribute to $\theta_{\beta }$ (Siewert and Voight 2017).

CLRT methods can also identify a skewed SFS. $B_{0,MAF}$ uses the folded SFS (as do NCD1 and $\beta^{(1)*}$), whereas $B_{0}$ uses the unfolded SFS (as does $\beta^{\left(1\right)}$). The key strength of these and other *B* statistics (Cheng and DeGiorgio 2020) is robustness to increasing window sizes, described in the following section. In summary, modern methods can be based on either summary statistics ($\beta^{\left(1\right)}$, $\beta^{(1)*}$, NCD1) or CLRTs ($B_{0}$, $B_{0,MAF}$), and they rely on the unfolded ($B_{0},\;\beta^{\left(1\right)}$) or folded (NCD1, $\beta^{(1)*}$, $B_{0,MAF}$) SFS (table 5).

### Methods to Identify Both Hallmark Signatures of LTBS

Identifying regions of the genome that simultaneously show an excess of polymorphism over divergence, and a SFS skewed to intermediate-frequency alleles, increases power and specificity. One of the first successful attempts to identify LTBS at the genome level in humans was that of Andrés et al. (2009), applying two separate tests ($MWU_{HIGH}$ and $HKA_{LOW}$) and considering as targets of LTBS only the genomic regions significant for both tests. This study established a principle that guided many recent methods: combining the different signatures expected under LTBS increases both power and specificity. Many modern methods do so, often as extensions of the methods outlined above.

The NCD2 statistic (tables 4 and 5) extends NCD1 by also counting substitutions between the tested population and an outgroup species. Substitutions are included in the statistic as alleles with frequency of one in the unfolded SFS (zero in the folded SFS), thus increasing the noncentral deviation from the *tf*. The intuition is that, due to old TMRCA, genomic regions under LTBS are expected to show a deficit of fixed differences relative to neutral expectations, and thus a lower NCD2 value. NCD2 is well-powered, simple to implement and interpret, fast to compute (Bitarello et al. 2018), and outperforms NCD1. Both versions of *NCD* are implemented in the R package balselr (table 4) and NCD2 is also implemented in MuteBaSS (Cheng and DeGiorgio 2019).

The $\beta^{\left(2\right)}$ statistic (Siewert and Voight 2020), implemented in BetaScan (tables 4 and 5) is an extension of its predecessor $\beta^{\left(1\right)}$, incorporating fixed differences and outperforming $\beta^{\left(1\right)}$ (Siewert and Voight 2020). $\beta^{\left(2\right)}$ introduces a new estimator to its statistic— $\theta_{D}$ —based on the number of fixed differences *D* between the ingroup population and the outgroup species, the divergence time between the two species *T*, the long-term effective population size ($N_{e}$) of the ingroup species, and the number of sampled chromosomes *n*: $\theta_{D}=\frac{D}{\frac{T}{2N_{e}}+\frac{1}{n}}$. Thus, $\beta^{\left(2\right)}={\hat{\theta }}_{\beta }-\theta_{D}$.

*T*_2_ (tables 4 and 5) explicitly models the spatial distribution of polymorphisms and substitutions—as described above for *T*_1_—and allele frequencies around a site under LTBS. This approach is very well-powered and, of all the methods described here, is the most complex one. The *T*_2_ test performs considerably better than *T*_1_ (DeGiorgio et al. 2014; Bitarello et al. 2018; Cheng and DeGiorgio 2020), confirming its effectiveness. However, computing *T*_2_ can be quite time-consuming. Thus, BALLET's authors (DeGiorgio et al. 2014) provide a number of simulated SFS to inform the likelihood calculations for humans. The provided SFS is extremely helpful although being specific to human demographic history limits the use of this approach for other species. This might explain why this method has not been as widely used, despite its high power.

Finally, $B_{2}$ and $B_{2,MAF}$ rely on a mixture model—described above for $B_{1}$ — but combine information about substitutions (as in $B_{1}$) and allele frequencies (as in $B_{0}$ and $B_{0,MAF}$) (table 5). A more recent implementation of the *B* statistics aims to simultaneously detect LTBS and positive selection, and these are implemented in the software BalLeRMix+ (Cheng and DeGiorgio 2021) (tables 4 and 5).

### Considerations on Choosing an LTBS Method

With so many methods available to identify LTBS, it is essential to compare their data requirements and the properties of each test regarding power, robustness, and types of selective regimes they can identify. Regarding the necessary data, some methods only require allele frequencies, while others need an outgroup sequence and knowledge of ancestral and derived states of alleles. To identify trPolym, high-quality sequence data for all species is required. Full sequencing is preferable for all analyses and allows the use of tests that cannot be implemented using genotyping data—for example, those that quantify the number of polymorphic sites (table 5 summarizes these data requirements). A key aspect to consider is statistical power, which depends on both the nature of the test and features of the data like demographic history and sampling strategy. While systematically comparing the power of these methods falls outside the scope of this review, we will comment on insights from published comparisons (DeGiorgio et al. 2014; Siewert and Voight 2017, 2020; Bitarello et al. 2018; Cheng and DeGiorgio 2019, 2020).

First, methods that combine two signatures of LTBS tend to be a more specific and powerful choice than those using a single signature (DeGiorgio et al. 2014; Siewert and Voight 2017, 2020; Bitarello et al. 2018; Cheng and DeGiorgio 2019, 2020) (fig. 2). Second, as a general rule, methods explicitly designed for BLS are more powerful than generalist ones such as Tajima's *D*, especially when the equilibrium frequency is not 0.5 (DeGiorgio et al. 2014; Siewert and Voight 2017, 2020; Bitarello et al. 2018). Third, there is always a loss of information in summary statistics. Therefore, CLRT methods tend to—but do not always—have more power than summary-based methods (DeGiorgio et al. 2014; Siewert and Voight 2017, 2020; Bitarello et al. 2018; Cheng and DeGiorgio 2019, 2020). The cost of running CLRT rather than summary-based methods is added computational time and difficulty in applying them to populations without well-defined demographic models.

Fourth, several important questions regarding the power and robustness of tests for LTBS remain to be addressed. New methods’ power and robustness analyses are often restricted to human demographic histories (DeGiorgio et al. 2014; Siewert and Voight 2017, 2020; Bitarello et al. 2018; Cheng and DeGiorgio 2019, 2020, 2021). Therefore, it will be necessary to evaluate the methods in the context of the histories of species of interest—although the relative power of the different methods would likely be similar across species.

Fifth, an important challenge concerns the size of the region being interrogated by each method (i.e., “window size”). Optimal window sizes differ among methods and may differ among species as a function of recombination rates. While the average width of an informative window for a given BLS timescale has been inferred for humans for most of the modern methods (Gao et al. 2015) as ranging between 1–3 Kb, species or genomic regions with different recombination rates may have different ideal window sizes. Power tends to decline with larger window sizes because the genomic segment with a signature represents a decreasing portion of the window. When increasing the window size from the optimal one to 25 Kb, HKA showed the greatest loss of power, followed by $\beta^{\left(2\right)}$, NCD2, $\beta^{\left(1\right)}$, $T_{1}$, $\beta^{(1)*}$, $T_{2}$ (in decreasing order of power lost) (Cheng and DeGiorgio 2020). The decline in power for *B* statistics with larger window sizes is much less pronounced, but for simple methods, using a very long (or very short) window will result in reduced power, making window length important. Thus, an advantage of the *B* statistics is that the method itself adjusts the window size to the data. Moreover, *B* statistics are computed substantially faster than the BALLET statistics $T_{1}$ and $T_{2}$, albeit considerably slower than summary statistics methods such as $\beta^{}$ and *NCD*.

Sixth, another source of discrepancy across studies is the criterion for choosing a representative window in simulations. Most studies only consider BLS simulations where the balanced polymorphism has not been lost. However, approaches vary considerably for the pairing of selected to neutral simulations. For instance, one could treat simulations with and without selection equally by centering windows on a fixed position (the site where the balanced polymorphism is inserted in simulations with selection). Alternatively, some studies require that each simulation with selection has a matching neutral simulation with the same target SNP frequency (e.g., Siewert and Voight 2017, 2020), whereas others take a more conservative approach and choose the most extreme value of the statistic for each simulation, whether neutral or with selection (Setter et al. 2020). These choices can generate differences in the reported performance of a given method, and there is no consensus on how they should be carried out. A related discrepancy across studies is whether a minimum number of informative sites is required when performing power analyses and the choice of performance metric used.

Finally, one significant limitation is that most, if not all, modern LTBS studies simulate LTBS using an overdominance model of BLS and consider several deterministic equilibrium frequencies (DeGiorgio et al. 2014; Siewert and Voight 2017, 2020; Bitarello et al. 2018). However, such equilibrium states are not guaranteed under other BLS regimes such as negative frequency dependence or fluctuating selection, as discussed above. Understanding the performance of methods under such scenarios and, potentially, devising methods able to distinguish between these mechanisms is a needed development in the field. Also, most studies only evaluate power under the assumption that a single site is under BLS, except for the *B* statistics (Cheng and DeGiorgio 2020).

Despite all these challenges in measuring power and comparing it across studies and methods, it is encouraging that current methods have high power to detect BLS, at least in the most commonly studied scenarios. It is important to carefully choose the best method for the studied species and apply it in a way that maximizes its power and considers potential artifacts (table 3), as discussed later. When these guidelines are followed, studies are able to correctly identify the main targets of BLS in most species.

## Methods to Detect ULTBS

Leffler et al. (2013) cleverly noted that ULTBS can generate short haplotypes shared between species. This signature can arise either because a neutral SNP is maintained in LD with a SNP under BLS, or because BLS favors a pair of epistatically interacting polymorphisms. Haplotype polymorphisms shared among species are convincing signatures of ULTBS, and are most likely in species or genomic regions with low effective recombination rate. Thus, identifying trPolym is arguably the best strategy to identify ULTBS. Nevertheless, attributing shared SNPs or haplotypes to BLS requires careful assessment of several confounding factors.

First, technical artifacts generate false shared polymorphisms, especially with short-read sequencing technologies where mis-mapping sequencing reads from unknown duplicates generates highly convincing (but false) shared polymorphisms (Teixeira et al. 2015). Second, most shared SNPs are due to recurrent mutation—often influenced by the context dependency of mutation rates (Hodgkinson and Eyre-Walker 2011). Thus, much effort to identify trans-species polymorphism is devoted to removing technical and biological artifacts, and while arduous, these efforts are critical. We recommend that identification of true trPolym requires at least: 1) establishing that due to high divergence among the species, the sharing of neutral variation is extremely unlikely (Gao et al. 2015); 2) identifying shared polymorphisms among the two (or more) genomes; 3) discarding recurrent mutations by showing the expected signatures of LTBS linked to the shared polymorphism, ideally in both species; and 4) discarding technical artifacts by validating the trPolym using alternative sequencing/genotyping techniques (Teixeira et al. 2015). We note that 4) is required even after 3) because, unfortunately, the technical artifacts that generate false shared polymorphism (e.g., unannotated genomic duplications) also generate false signatures of ULTBS.

Thankfully, recent methods can identify the narrow-ranged signatures of ULTBS around trPolym (Cheng and DeGiorgio 2019) (fig. 2). Modified versions of HKA, NCD2, $T_{1}$, and $T_{2}$ — $HKA_{trans},NCD2_{trans},T_{1,trans},\;T_{2,trans}\;$ (Cheng and DeGiorgio 2019)—, implemented in MuteBaSS (table 4) (Cheng and DeGiorgio 2019) can detect signatures of trans-species polymorphism even when the shared polymorphisms are not present. The main difference between these modified versions and their original predecessors is that they use population data for both ingroup and outgroup species (table 5). That way, polymorphic sites, fixed differences, and shared polymorphic sites are more clearly picked up. Moreover, these methods can be extended to an arbitrary number of species rather than being restricted to two species comparisons. While they were proposed with the intent of detecting trans-species scale BLS, they can also be used for LTBS.

## Methods to Detect RBS

RBS has not been studied as extensively as LTBS and ULTBS because unequivocally identifying its signature in genomes is challenging—it is often essentially indistinguishable from signatures of recent, ongoing, or incomplete selective sweeps. Nevertheless, a few approaches show promising results and we describe them here. In our view, the ability to distinguish sweeps due to positive selection and those due to RBS is one of the most exciting current developments in the field.

RBS can generate an excess of identity by descent, an increased density of singleton variants (Field et al. 2016), or a decrease in the overall level of genetic differentiation among populations, usually measured with $F_{ST}$ (Lewontin and Krakauer 1973). BLS is likely to be shared among populations especially when selective pressures are not heavily dependent on the environment, such as in sexual antagonism (Connallon and Clark 2014). However, it is not straightforward to identify candidates with low $F_{ST}$, partly because many species have, as humans do, low $F_{ST}$ as a neutral baseline.

The $T_{sel}$ method (Hunter-Zinck and Clark 2015) (table 4) uses pairwise TMRCA estimates to approximate the genealogies at a nonrecombining locus. Anomalies generated by several modes of natural selection can be detected using this method, and the authors reported that the power to detect RBS (on the scale of about 10,000 years) in an overdominance model was higher than that for the HKA test (Hunter-Zinck and Clark 2015).

In pioneering work using multilayered neural networks for complex population genetic modeling, Sheehan and Song (2016) established the promise of machine learning (ML) tools for joint inference of natural selection and demography. ML-based approaches can learn from training data (typically, simulations of several different demographic and selective parameters), use hundreds of available summary statistics, and then rely on the trained network to infer demographic and selective events from new data. Sheehan and Song (2016) provided proof of concept that this could be done to untangle demographic and selective signatures in *Drosophila*. An appeal of this approach is its potential to correctly classify loci in different selection models (Sheehan and Song 2016; Isildak et al. 2021) even when we fail to predict (or even to understand) the theoretical basis for their different genomic signatures.

Isildak et al. (2021) applied this foundational framework to study BLS, using convolutional neural networks to untangle demographic from selective events, positive from BLS, and RBS from incomplete sweeps. Remarkably, they found accuracy of ∼70% to differentiate between BLS and selective sweeps on a recent timescale for humans (20,000 years). This seems like a promising avenue for future research, and this approach can be used for other species and is available as the software BaSe (table 4).

## The Genomic Prevalence of Balancing Selection

A central question in evolutionary biology is how much variation is maintained by natural selection. Although it is well documented that BLS plays a prominent role in maintaining specific polymorphisms, its prevalence, and degree of contribution to overall levels of genetic and phenotypic diversity in natural populations remain debated. Our limited power to identify BLS signatures at ultra long and recent timescales hampers our ability to answer this question, but we are starting to have some estimates.

ULTBS and trans-species polymorphisms are exceedingly rare outside loci such as the MHC and ABO (Leffler et al. 2013; Gao et al. 2015; Teixeira et al. 2015). LTBS is also uncommon, although likely more common than previously thought. Bitarello et al. (2018) estimated that between 0.37% and 0.6% of the analyzed human genome and between 1% and 8% of 18,633 analyzed human protein-coding genes have signatures of LTBS incompatible with neutral expectations under a realistic human demographic model. An alternative approach is to quantify BLS's genomic signature without identifying individual loci, thus bypassing the difficulties in identifying individual evolutionary events.

In the McDonald–Kretiman (MK) framework (McDonald and Kreitman 1991), an excess of nonsynonymous substitutions across species, when compared with nonsynonymous polymorphism within populations (and contrasted with synonymous variants as a proxy for neutrality) can, under some conditions, be attributed to the effects of positive selection. Soni et al. (2022) extended the MK philosophy to BLS. Under their model, an accumulation of (primarily old) nonsynonymous polymorphisms shared across populations compared with those private to one population (primarily recent) indicates BLS that maintains old, functional polymorphisms in populations. The method is ideal for quantifying genome-wide BLS effects but has little power for individual genes, as it relies on genome-wide polymorphism counts. The method is potentially sensitive to selection at any time before population differentiation, including periods that are virtually inaccessible to other methods. Soni et al. (2022) estimate that in humans, 2–4% of nonsynonymous polymorphisms shared between African and non-African populations (200–400 SNPs) are maintained by BLS. This estimate depends on several assumptions and may not be precise, but the inference of substantial numbers of nonsynonymous SNPs under BLS is broadly in agreement with Bitarello et al. (2018), who found targets of LTBS are enriched in nonsynonymous SNPs, and estimated between 223–913 and 180–689 nonsynonymous SNPs are within BLS targets in African and European genomes, respectively.

In principle, recent and transient BLS could be even more common, although we do not have reliable estimates quite yet (Messer et al. 2016). Theoretical population genetics predicts that short-lived heterozygote advantage may be common in the path to new adaptations and, thus, relatively common in natural populations (Sellis et al. 2011). If BLS is as prevalent in noncoding genomic regions as in exons, for example due to selection on regulatory regions where the selective pressures and favored variants differ across tissues (Wegmann et al. 2008)—the number of balanced polymorphisms could be in the thousands. If, as predicted by theory, very recent and transient BLS is more common than LTBS, the number of polymorphisms maintained by BLS—and the magnitude of phenotypic diversity maintained by natural selection—could be orders of magnitude higher than current estimates suggest (Messer et al. 2016). In any case, these studies suggest that BLS may be prevalent in human populations—and if so, likely also in other species.

## Implications of Balancing Selection to Evolutionary Research

What is the importance of identifying genes and genomic regions under BLS? Research on BLS is intimately connected to other areas of evolutionary research, providing insights into evolutionary processes. Identifying selected loci is thus a starting point to address many other questions.

Researchers have long recognized the importance of host–pathogen co-evolutionary dynamics, and BLS has been a central theme in theoretical modeling and empirical analyses of host–pathogen interactions (Ebert and Fields 2020). BLS naturally arises in this context because hosts adapt to a constantly evolving counterpart, the pathogen, while pathogens likewise are under selection and adapt to the host. Examples include the geographic patterns of adaptation in *Leishmania* (Grace et al. 2021) and studies of the malaria parasite *Plasmodium falciparum*, which aim to identify the stage in the life-cycle that expresses genes under strong BLS, as well as the degree of expression (Amambua-Ngwa et al. 2012). Of note, a study of shared BLS between humans and bonobos (Cheng and DeGiorgio 2020) found a top candidate region between the *FREM3* and *GYPE* genes, both of which had been previously described by Leffler et al. (2013) as BLS targets related to blood group phenotype and protection against the malaria parasite *P. falciparum*. On the pathogen side of host–pathogen interactions, knowledge of pathogen proteins that are under BLS provides crucial information for vaccine development, prioritizing pathogen surface proteins to which the host immune system responds.

Increasingly BLS is considered in studies of complex traits, connecting molecular and functional variation. In a study of the wildflower *Boechera stricta*, Carley et al. (2021) used a combination of transplantation experiments and genomic analyses and suggested that BLS in genes underlying leaf chemical response arises due to pleiotropic effects of genes underlying chemical biosynthesis on two different phenotypes: defense from herbivory and response to drought. Another study addressing an essential phenotypic trait examined plumage polymorphism in the Gouldian finch (Kim et al. 2019). A locus with a substantial effect on the phenotype was identified and shown to have signatures of strong BLS, once again with pleiotropic effects proposed as a likely underlying mechanism. In this case, the conflict between genetic contribution to the attractiveness of males was counterbalanced by decreased survival. A similar pattern was documented in Soay sheep, where a variant contributing to large horn size is advantageous in intrasexual competition but disadvantageous to survival (Johnston et al. 2013). Pleiotropy also appears to play a role in the pacific oyster *Crassostrea gigas*, where an experimental assessment of life-history stages showed heterogeneous selection pressures on different stages in the life-cycle (Durland et al. 2021). Overall, pleiotropy is seemingly a recurrent and essential biological property of loci showing a BLS signature.

While many studies designed to identify selection trace the unit of selection to specific genes, there is increasing interest in understanding how BLS shapes polymorphism in copy number variants, inversions, and deletions (Fijarczyk and Babik 2015). Although methodological aspects of studying BLS in these types of structural variants fall outside the scope of this review, some examples highlight their biological relevance. In the white-throated sparrow, plumage polymorphism segregates with variation in chromosome structure, a large inversion spanning 15 genes, and maintained in a polymorphic state at intermediate frequencies (Jeong et al. 2022). Within these inversions, there are signatures of directional selection and BLS, whereas the maintenance of the inversions themselves shows evidence of BLS. Inversions generate recombinational products that are usually unviable (due to defective chromosome segregation), causing the population recombination to be substantially suppressed and contributing to the accumulation of deleterious mutations within inversions. In this context, BLS would maintain variability in a region prone to losing variation due to reduced recombination. Selection on inversions and other forms of structural variation is only beginning to be addressed using genome-wide data because tools for identifying variation of this nature are relatively new. An indication of the importance of BLS for structural variation also comes from a study in humans, which found an enrichment of BLS signatures among polymorphic inversions (Giner-Delgado et al. 2019) and another study suggesting that some ancient human deletions carry signatures of LTBS (Aqil et al. 2023).

While inversions represent a region of low recombination within an otherwise recombining genome, selfing species show a genome-wide effect of suppressed population-level recombination. Glémin (2021) recently developed a theoretical framework showing how selfing alters the effectiveness of different types of BLS and that frequency-dependent regimes—differently from overdominant-type regimes—are not substantially affected by selfing. In *C. elegans—*where selfing is an important mode of reproduction—Lee et al. (2021) found that hyperdivergent haplotypes were maintained in a background otherwise characterized by low variation. Because genes in the divergent genomic regions are enriched for functions related to sensory perception, pathogen response, and xenobiotic stress response, the authors suggest that BLS may be playing a crucial role in reversing the detrimental evolutionary effects of selfing.

The relationship between BLS and population structure is a further thread of theoretical and empirical interest. The canonical view is that BLS maintains shared alleles among populations, resulting in low differentiation among them (Lewontin and Krakauer 1973). However, other scenarios are theoretically plausible, including one in which distinct populations are locally adapted to different combinations of advantageous alleles so that they are individually under BLS, but with different frequencies of the selected alleles (Maróstica et al. 2022). Yet another scenario is one in which a species occupies a large range, so different populations have distinct locally adapted variants. While the individual populations may carry signatures of local adaptation, if the species as a whole was for some reason analyzed as a single unit, the signature would erroneously resemble that of BLS. With local adaptation on an allele previously under BLS, BLS signatures will not be shared across all populations, with some showing noncanonical signatures of positive selection that can nevertheless be identified (de Filippo et al. 2016). Finally, it is worth noting that local adaptation followed by extensive gene-flow may generate signatures that resemble in some ways those of BLS at the level of the species as a whole and the individual populations.

In humans, genome-wide BLS studies have also provided insights into functional categories with multiple genes under selection, helping us understand the biological processes driving BLS. Among recent studies, there is a recurrent overrepresentation of immune-related genes among LTBS candidates (Andrés et al. 2009; DeGiorgio et al. 2014; Siewert and Voight 2017, 2020; Bitarello et al. 2018; Cheng and DeGiorgio 2019, 2020), an increased number of nonsynonymous sites among candidate regions (Bitarello et al. 2018), overrepresentation of genes with mono-allelic expression in humans (Savova et al. 2016; Bitarello et al. 2018), and overrepresentation of cell-surface receptor genes (Andrés et al. 2009; DeGiorgio et al. 2014; Key et al. 2014; Bitarello et al. 2018). Other findings are somewhat contradictory: some studies suggest most LTBS targets are noncoding (Leffler et al. 2013) while others fail to recapitulate this result (e.g., Bitarello et al. 2018).

## The Road Ahead

The integration of large genomic datasets and ever-evolving neutrality tests has substantially improved our understanding of the influence of BLS in genomes. Progress has been remarkable, especially for humans, showing that BLS is an evolutionary force that cannot be ignored. However, a successful future investigation of BLS will require efforts on at least five fronts.

First, as discussed throughout this review, a central challenge is to account for alternative demographic and evolutionary processes that generate similar signatures, and processes that reduce the power of existing tests. Research in the field has acknowledged this—all recent methods are designed and presented accounting for the possible impact of demographic history on the findings (e.g., Siewert and Voight 2017, 2020; Bitarello et al. 2018, Cheng and DeGiorgio 2019, 2020, 2021). It follows that, as with all other population genetic analysis, the better we understand the demographic history of a population, the more accurate inferences of BLS will be. Incorporating these into neutral simulations is simpler in well-studied species, but will be challenging for those with less demographic information. The possibility of generating complex demographic histories using simulations (e.g., Hoban et al. 2012) will allow many demographic scenarios to be explored. An increasingly attractive alternative is to use methods that simultaneously estimate selective and demographic parameters, as can be done, in principle, using ABC or ML.

Second, background selection (BGS) is also important, particularly for species with a large effective population size. BGS can reduce genetic diversity, thus shifting the null expectations. Thus, accounting for it will increase the power to identify targets of BLS (e.g., Comeron 2014), while ignoring it makes BLS tests conservative. Nevertheless, the immense realm of possible demographic scenarios in combination with many additional factors (e.g., variation in mutation and recombination rates, among others), makes a complete exploration of parameter space unrealistic. In light of this, formulating research questions in the form of more narrowly defined hypothesis tests provides an attractive alternative. For example, rather than asking “Is there evidence of BLS” at a locus, one could potentially ask whether it is possible to rule out other factors—including nonselective ones—to realistically generate genomic regions with the observed signatures of BLS. For example, one could ask: “How high would levels of introgression have to be, in order to generate the proportion of sites we see with unusually extreme SFS”? “Do candidate loci show the additional signatures expected under such introgression event?” These questions are not directly about the prevalence or targets of selection, but they provide a means of ruling out certain alternatives.

Third, we believe the community must adhere to strict data processing and filtering. Common technical artifacts of next-generation sequencing, especially mis-mapping of reads, mimic BLS signatures. Thus, before analyzing a genome, rigorous filtering to remove unannotated segmental duplications, unannotated paralogs, and pseudogenes from the datasets—far from a trivial task, especially in organisms with poor-quality genomes or genome annotation, arguably among the most interesting ones (e.g., endangered species). We suggest that all genomic BLS studies must include stringent filters of the genomic data and detailed checks of the candidates to limit such issues—see for example, Bitarello et al. (2018) and Cheng and DeGiorgio (2020).

Fourth, and related, the very outcome of BLS—increased polymorphism—generates technical challenges in obtaining reliable genetic data. For example, in HLA genes, the combination of high polymorphism and extensive paralogy results in many reads obtained in next-generation sequencing assays being mapped to the incorrect locus, or not mapping to any locus for the available reference genome (Meyer et al. 2018). This problem can be circumvented by using alternative mapping strategies—for example abandoning single reference genomes, using variation-aware approaches, or employing long-read sequencing (Meyer et al. 2018). If these strategies are not employed, we may end up not having appropriate data to test for BLS in precisely the regions where this process is particularly important.

Fifth, we see the need for developing and validating tests capable of detecting BLS on different timescales as paramount. Even with well-curated datasets, identifying the genomic BLS signatures remains a challenge for diverse selective regimes: old selection (with its narrow genomic signatures and rare occurrence), very recent selection (with signatures that resemble those of other types of selection), and selective forces that depart from classical signatures (such as rare allele advantage). Nevertheless, as we have reviewed here, the last few years have brought tremendous advances, with increasingly sophisticated approaches designed to identify selection at various timescales. It is worth noting that because of the long-term nature of many balanced loci, multiple selective mechanisms are likely to co-occur in a given locus, at the same or different timescales. The interpretation of such complex signatures can be challenging but also creates opportunities. For example, accounting for the action of LTBS in a locus helps identify targets of local adaptation from standing variation (de Filippo et al. 2016).

Finally, a formidable outstanding challenge in studying BLS will be making biologically sound inferences about the underlying biological processes of selected genes or genomic regions. An important reason is that the imperfect annotation of genomes means that we often do not know the function of most SNPs—or even genes. Linking patterns of genetic variation to phenotype and putative selective forces is even more challenging. The dual complexity of distinguishing among different BLS mechanisms based on genomic signatures alone and the challenges of biological interpretation of genomic signatures of natural selection, in general, makes the biological interpretation of BLS candidate loci challenging. Still, combining information at the genomic, phenotypic, and environmental levels has allowed us to discover and understand fascinating instances of BLS.

## Data Availability

No new data were generated or analyzed in support of this article.
